# Combination of Carbonate of Iron with Sulphate of Quinine in Intermittent Fever

**Published:** 1846-10

**Authors:** 


20. *Combination of Carbonate of Iron with Sulphate of Quinine in Intermittent Fever.*—Prof. LIPPICH, of Padua, recommends the addition of the carbonate of iron to the sulphate of quinine in the treatment of periodical fevers. The following is his formula:—

| | |
|---|---|
| Carbonate of iron, - - | One gramme. |
| Sulphate of quinine, - - - | One gramme. |
| Extract of taraxacum - - | q. s. |

To be made into a mass of proper consistency and divided into thirty pills, two of which are to be taken every two hours. The carbonate of iron may be gradually increased to two grammes.—*Gaz. Méd. de Paris*, in *Med. News.*

